# Decreased liver-to-spleen ratio in low-dose computed tomography as a biomarker of fatty liver disease reflects risk for myocardial ischaemia

**DOI:** 10.1093/ehjimp/qyad016

**Published:** 2023-08-10

**Authors:** A Hokkanen, H Hämäläinen, T M Laitinen, T P Laitinen

**Affiliations:** Department of Clinical Physiology and Nuclear Medicine, Kuopio University Hospital, Puijonlaaksontie 2, 70200 Kuopio, Finland; School of Medicine, University College Cork, Ireland; Department of Clinical Physiology and Nuclear Medicine, Kuopio University Hospital, Puijonlaaksontie 2, 70200 Kuopio, Finland; Department of Clinical Physiology and Nuclear Medicine, Kuopio University Hospital, Puijonlaaksontie 2, 70200 Kuopio, Finland; Department of Clinical Physiology and Nuclear Medicine, Kuopio University Hospital, Puijonlaaksontie 2, 70200 Kuopio, Finland; Institute of Clinical Medicine, University of Eastern Finland, Kuopio, Finland

**Keywords:** fatty liver disease, hepatic steatosis, myocardial perfusion, ischaemia, left ventricular function, low-dose computed tomography

## Abstract

**Aims:**

A strong association between fatty liver disease (FLD) and coronary artery disease is consistently reported. Our aim was to evaluate whether FLD diagnosed using low-dose non-contrast computed tomography (LDCT), as a by-product of myocardial perfusion imaging (MPI), is associated with myocardial ischaemia or left ventricular function parameters.

**Methods and results:**

We analysed 742 patients who had undergone MPI using single photon emission computed tomography (SPECT) and LDCT. A liver-to-spleen ratio (in Hounsfield units) of <1 was defined as FLD. Myocardial ischaemia was defined as a summed difference score (SDS) ≥3. Left ventricular size and systolic function were assessed from the electrocardiogram-gated SPECT. FLD patients were younger (63 vs. 68 years) and had a higher body mass index (34.6 vs. 29.0 kg/m^2^) and a higher SDS (2.65 vs. 1.63), *P* < 0.001 for all. Independently of several possible confounding factors including traditional risk factors, patients with FLD had a 1.70-fold risk of ischaemia (95% confidence interval 1.11–2.58, *P* = 0.014). Left ventricular end-diastolic volume (109 vs. 109 mL) and ejection fraction (61 vs. 61%) were comparable in those with and without FLD (non-significant for both).

**Conclusions:**

With the help of LDCT, it is possible to identify FLD, which is associated with an increased risk of myocardial ischaemia. Therefore, evaluation of FLD from LDCT is recommended along with MPI.

## Lay Summary

Fatty liver disease is the most common liver disease, affecting a quarter of the population worldwide. It is connected to diabetes and high blood pressure as well as to overweight and obesity. Computed tomography is a common diagnostic method. Its downside is that it exposes patients to radiation. Images can be taken with lower doses of radiation, but these images are less clear and often cannot be used for diagnosis. Low-dose computed tomography is often used with other methods of medical imaging, especially for the heart and lungs. This study shows that fatty liver disease can be diagnosed as a free by-product of low-dose computed tomography images gathered as part of other diagnostic imaging. We also show that fatty liver disease diagnosed from these images is connected to reduced blood flow of heart muscle. Based on the findings, we recommend that these images be scanned for fatty liver disease to identify patients who are at higher risk of heart disease.

## Graphical Abstract

**Graphical abstract:**
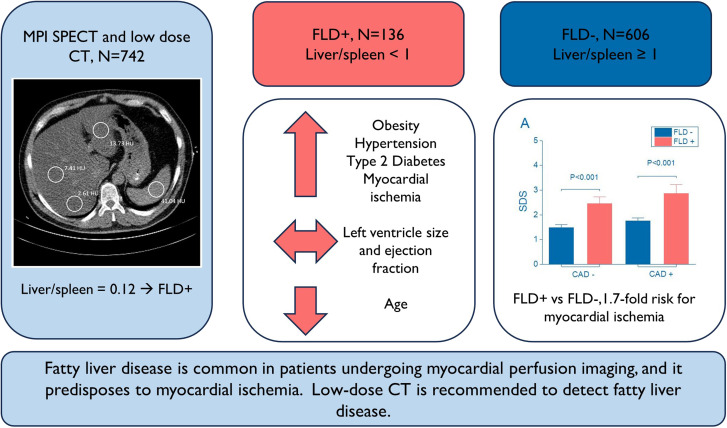

## Introduction

Computed tomography (CT) is widely used in almost all specialties for a large variety of indications, whether for diagnostic purposes or to assist other imaging and diagnostic modalities. Low-dose CT (LDCT) is not typically used for diagnostic purposes as it has lower image quality, though it is commonly used for attenuation correction in processes such as myocardial perfusion imaging (MPI) single photon emission CT (SPECT). Nevertheless, these images can and should be analysed for any findings.^1^ A study by Zadro *et al.*^2^ reported minor or major incidental extra-cardiac findings in 69.2% of patients undergoing MPI SPECT, with 53.3% of the major findings having been previously unknown. Among the most prevalent minor findings was fatty liver disease (FLD), with a prevalence of 5.9%.^3^ Although not the most accurate method of diagnosing FLD, LDCT images have shown high sensitivity and specificity of 86.3 and 81.4%, respectively,^4^ as well as a good test–retest and inter-observer variability in the diagnosis of FLD.^5^

With a global prevalence of 25%, FLD is the most common liver disease.^6^ FLD has multiple risk factors in common with cardiovascular diseases (CVDs); this is known as the liver–heart axis.^7^ Furthermore, FLD is a significant and independent risk factor for CVDs as well as for cardiac structural and functional changes.^8,9^ Since FLD is strongly associated with metabolic syndrome and its components, such as insulin resistance, hypertension, and dyslipidaemia,^8^ studying the links between FLD and CVDs without the effect of these risk factors can be challenging. Regardless of external risk factors, patients with FLD are at higher risk for coronary artery disease (CAD), myocardial infarction, and atrial fibrillation compared with patients without FLD.^7,10^

Atherosclerotic changes, especially in the coronary arteries,^7,11^ are associated with or can directly cause changes in myocardial perfusion. Even though extensive research has been carried out on the associations between FLD and CVD, little is known about the sub-clinical and clinical effects of FLD on myocardial perfusion. Additionally, studies have reported contradictory results; for instance, FLD has been shown to be associated with reduced left ventricle ejection fraction (LVEF),^12–14^ while other findings have indicated the contrary.^15–18^

The aim of this study was to investigate whether LDCT in the MPI SPECT/CT protocol is sufficiently accurate in identifying high-risk individuals with FLD and whether FLD contributes to cardiac disorders diagnosed using MPI.

## Methods

### Study population

The study population consisted of 936 patients who underwent MPI SPECT and LDCT at Kuopio University Hospital. Of these, 194 had a normal stress phase or declined the rest phase. For 742 patients, both stress and rest phase scans were performed. The protocol for the study was approved by the Ethics Committee of the Northern Savo Hospital District.

Patient information and medical history were collected from medical records. Basic information such as age, weight, and height (with an accuracy of 1 kg and 1 cm, respectively) was available for the entire population. Medication information was available for 734 patients. History of comorbidities was known for all patients. Perfusion scores were also available for the entire population, while volume parameters were available for 730 patients. Information about alcohol consumption was incomplete, as it was not systematically mentioned in medical records for all participants.

### Imaging protocol

MPI consisted of separate stress and rest scans using a 1-day protocol with at least 3 h between the two scans. Stress was induced using adenosine combined with low-intensity exercise (starting at 20 W and raised to 60 W for 4 min) when possible. During the pharmacological stress, 300 MBq ^99m^Tc-tetrofosmin was administered intravenously, and imaging was performed 30–45 min after the pharmacological stress provocation. In the rest phase, 700 MBq ^99m^Tc-tetrofosmin was administered. The rest scan was performed ∼45 min after administering the tracer. All patients were required to avoid coffee, tea, and other caffeine-containing products for at least 12 h before MPI.

Imaging was performed in a supine position using a Philips Precedence dual-head SPECT/CT hybrid camera with a six-slice CT scanner (Philips Medical Systems, Bothell, WA, USA). The detectors were in a 90°configuration, and 180° body contour orbits were used. The data were acquired in 64 projections with a 128 × 128 matrix, with each angle measured for 25 s (patient weight <100 kg) or 30 s (patient weight ≥100 kg). HERMES Hybrid Recon Cardiology (Hermes Medical Solutions AB, Stockholm, Sweden) was used for the reconstruction of the images, and QPS/QGS 2012 (Cedars-Sinai Medical Center, Los Angeles, CA, USA) software was used for perfusion and ventricular function quantification. LDCT (140 kV, 35–72 mA, and 20–40 mAs) was part of the protocol for attenuation correction and was performed in both imaging phases.

### MPI analysis

The quantitative summed rest score (SRS), summed stress score (SSS), and summed difference score (SDS) were calculated using QPS software and a 17-segment model of the left ventricle as described by Germano *et al.*^19^ Each segment was given a score between 0 and 4 based on tracer uptake (0, normal uptake; 1, mildly reduced uptake; 2, moderately reduced uptake; 3, severely reduced uptake; or 4, absent uptake). Scoring was performed automatically by QPS/QGS 2012 software (Cedars-Sinai Medical Center) using our own normal database as a reference.^20^ The SDS was calculated by subtracting the SRS from the SSS. An SDS ≥ 3 was defined as myocardial ischaemia. Co-registration of rest and stress images and voxel-by-voxel estimation of differences as described by Slomka *et al.*^21^ were also used in quantifying ischaemia percentage (ischaemia%).^22^

Values for left ventricle size and function were automatically calculated from the electrocardiogram (ECG)-gated SPECT. From the 16-time bins, QGS software was able to calculate end-diastolic volume (EDV), end-systolic volume (ESV), and ejection fraction (EF), based on left ventricular size variation along the cardiac cycle.^20^

### CT analysis

The MPI imaging protocol included an LDCT scan spanning the upper abdomen, which was used to diagnose FLD. This was performed as described by Kerut *et al.*^5,23^ We measured the hepatic and splenic attenuation [in Hounsfield units (HUs)] of four regions of interest (ROIs). One ROI was measured in the spleen and the other three in the liver: posterior right lobe, anterior right lobe, and left lobe. All ROIs were at least 100 mm^2^ in size, and heterogeneous areas were avoided in the measurements. Based on the measurements, we calculated a liver-to-spleen ratio (L/S) by dividing the average of the HU values of the two ROIs in the right lobe of the liver by the HU value of the spleen. The process was explained in a previous study.^5^ A threshold L/S of <1 indicates FLD.

In a previous study,^4^ CT has exhibited high specificity and sensitivity, 86.3 and 81.4%, respectively, as well as a high test–retest repeatability {Concordance correlation coefficient 0.877, Intraclass correlation coefficient 0.878 [95% confidence interval (CI) 0.847–0.903]}.^5^

### Statistical methods

The calculated L/S ratios were used to divide the population into two groups. Patients with L/S < 1 were regarded as patients with FLD (FLD+), and those with L/S ≥ 1 were regarded as normal (FLD−). For continuous variables, a comparison between the two groups was performed using the independent samples *t*-test. A χ^2^ test was applied to sets of categorical data. We analysed strength of association between FLD and myocardial ischaemia with careful adjustments for potential confounding factors. We included age, sex, overweight/obesity, hypertension, dyslipidaemia, Type 2 diabetes, and previous diagnosis of CAD as potential confounders. We estimated a multivariable logistic regression model between the potential confounders and FLD in order to assess individual propensity scores for the presence of FLD. Then, we employed logistic regression to examine the association between FLD and myocardial ischaemia (SDS ≥ 3 vs. SDS < 3). Firstly, FLD was included as the only covariate (Model 1). Confounding was thereafter controlled for by including the propensity score as a covariate (Model 2). To adjust the statistical significance for age and sex, we used analysis of covariance, and logistic regression was used to calculate the odds ratio. A *P*-value below 0.05 was considered statistically significant. SPSS software (IBM SPSS Statistics, 2013, version 22) was used for statistical analysis of the data.

## Results

Clinical characteristics of the study population are shown in *Table 1*. Our study population consisted of 742 patients (406 male, 54.8%), with a mean age of 67 years. The prevalence of FLD was 18.3%. Participants in the FLD+ group were younger than those in the FLD− group. Sex distribution was comparable between the groups. Compared with the FLD− group, participants in the FLD+ group had significantly higher body weight (99 vs. 81 kg), body mass index (BMI) (34.6 vs. 29.0 kg/m^2^), and diastolic blood pressure (80 vs. 76 mmHg).

**Table 1:** Clinical characteristics of the study population

	Pooled population (*n* = 742)	FLD− (*n* = 606)	FLD+ (*n* = 136)	*P*-value (FLD− vs. FLD+, age- and sex-adjusted)
Age (years)	67 ± 11	68 ± 11	63 ± 10	**<0.001**
Male	406 (54.8%)	322 (53.2%)	84 (61.8%)	0.255
Weight (kg)	84 ± 20	81 ± 17	99 ± 24	**<0**.**001**
Height (cm)	167 ± 10	167 ± 10	169 ± 9	0.998
Body mass index (kg/m^2^)	30.0 ± 6.4	29.0 ± 5.5	34.6 ± 7.9	**<0**.**001**
Systolic blood pressure (mmHg)	146 ± 23	146 ± 24	146 ± 22	0.551
Diastolic blood pressure (mmHg)	77 ± 11	76 ± 11	80 ± 12	**0**.**010**
Heart rate (bpm)	68 ± 12	67 ± 12	72 ± 13	**<0**.**001**
Beta-blocker	532 (72.5%)	434 (72.2%)	98 (73.7%)	0.328
ACE/AT	454 (61.9%)	362 (60.2%)	92 (69.2%)	**0**.**026**
Calcium blocker	213 (29.0%)	169 (28.1%)	44 (33.1%)	0.125
Nitrate	287 (39.1%)	240 (39.9%)	47 (35.3%)	0.754
Diuretic	261 (35.6%)	211 (35.1%)	50 (37.6%)	0.112
Antiarrhythmic	13 (1.8%)	12 (2.0%)	1 (0.8%)	0.446
Digoxin	35 (4.8%)	28 (4.7%)	7 (5.3%)	0.443
Cholesterol medication	501 (68.3%)	418 (69.6%)	83 (62.4%)	0.176
Acetyl salicylic acid	420 (57.2%)	343 (57.1%)	77 (57.9%)	0.509
Diabetes medication	187 (25.5%)	125 (20.8%)	62 (46.6%)	**<0**.**001**
Anticoagulant	264 (36.0%)	229 (38.1%)	35 (26.3%)	0.061
Overweight (BMI ≥ 25 kg/m^2^)	594 (80.8%)	468 (77.9%)	126 (94%)	**<0**.**001**
Obese (BMI ≥ 30 kg/m^2^)	309 (42.0%)	212 (35.3%)	97 (72.4%)	**<0**.**001**
Hypertension	554 (74.7%)	437 (72.1%)	117 (86.0%)	**<0**.**001**
Dyslipidaemia	427 (57.5%)	340 (56.1%)	87 (64.0%)	0.125
MI	154 (20.8%)	133 (21.9%)	21 (15.4%)	0.061
PCI	140 (18.9%)	114 (18.8%)	26 (19.1%)	0.950
CABG	140 (18.9%)	123 (20.3%)	17 (12.5%)	0.117
Diabetes	230 (31.0%)	160 (26.4%)	70 (51.5%)	**<0**.**001**
Pulmonary disease	208 (28.0%)	164 (27.1%)	55 (32.4%)	0.230
CAD	386 (52.0%)	323 (53.3%)	63 (46.3%)	0.595

Values are mean ± SD or number of cases (%). Statistically significant *P*-values in bold.

ACE/AT, angiotensin-converting enzyme inhibitor or angiotensin receptor antagonist; MI, myocardial infarction; PCI, percutaneous coronary intervention; CABG, coronary artery bypass graft surgery.

**Figure 1:**
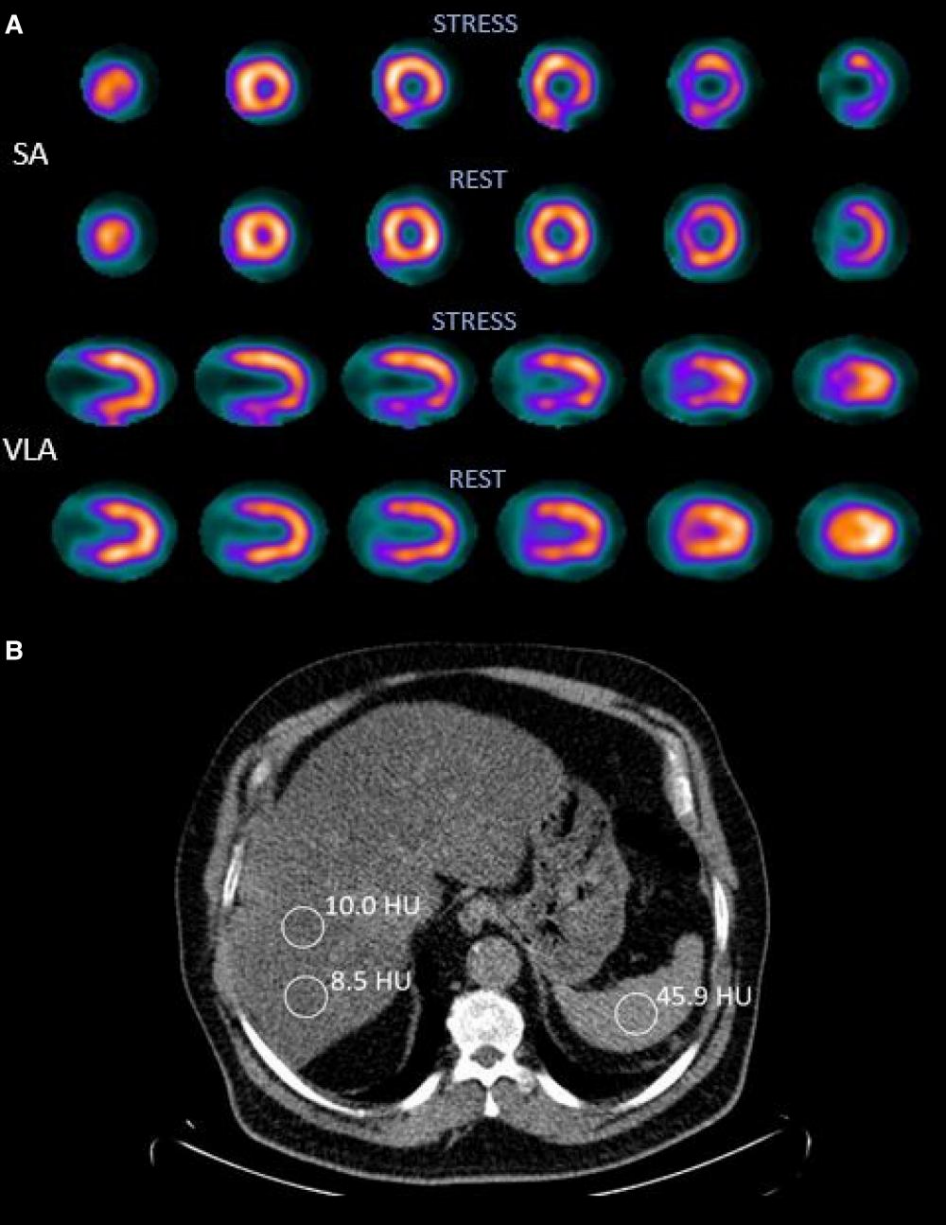
Myocardial perfusion (*A*) and LDCT (*B*) images of a patient with FLD. Short-axis (SA) and vertical long-axis (VLA) slices from MPI of a 69-year-old man who had hypertension, dyslipidaemia, and Type 2 diabetes. Note reversible myocardial ischaemia in the inferior wall of the myocardium. The L/S in a LDCT was 0.40 (<1.0 indicates FLD).

Hypertension and diabetes were statistically significantly more prevalent in the FLD+ group as compared with the FLD− group, while other comorbidities, such as previously diagnosed CAD, respiratory disease, and previous MI, had comparable prevalence between the groups. As diabetes and hypertension were more prevalent in FLD+, they were also statistically significantly more likely to be using angiotensin-converting enzyme inhibitor or angiotensin receptor antagonist or diabetes medication (69.2 vs. 60.2% and 46.6 vs. 20.8%, respectively) (*Figure 1* and *Table 1*).

**Figure 2:**
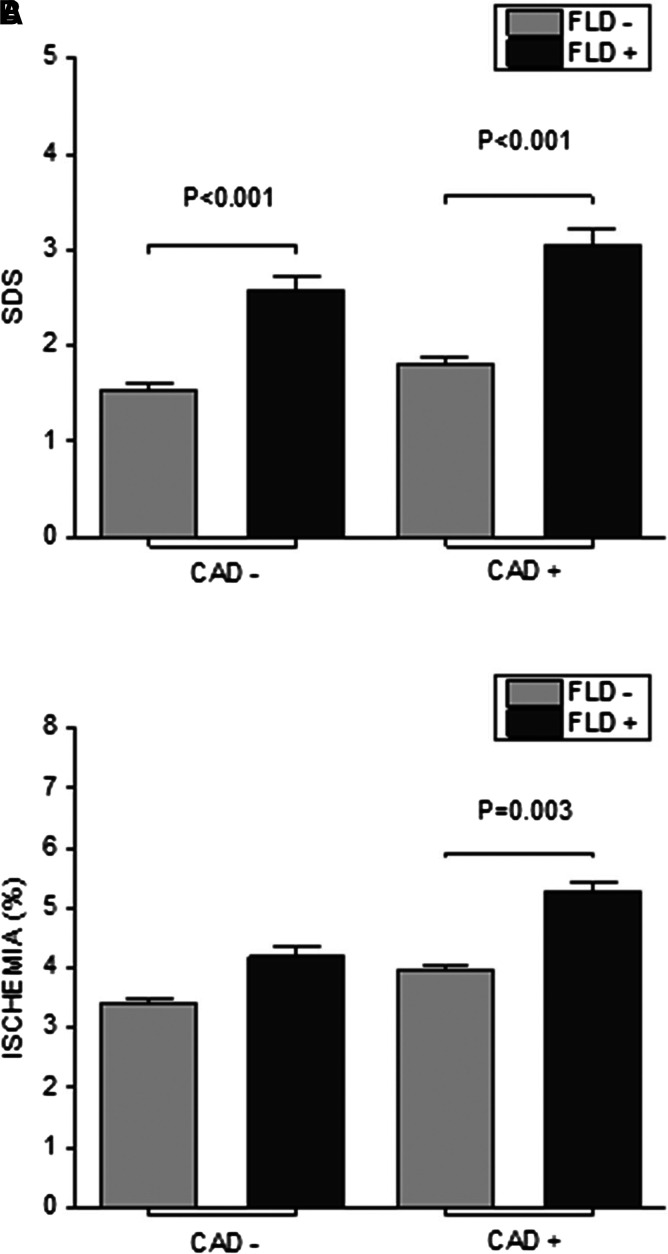
Myocardial perfusion and coronary artery disease. SDS (*A*) and ischaemia% (*B*) of patients with (CAD+) and without (CAD−) coronary artery disease. FLD causes an increase in both SDS and ischaemia% independently of CAD status.

The patients defined as FLD+ had a significantly higher ischaemia%, 4.54 compared with 3.63 for the FLD− group, *P* = 0.001 (*Figure 1* and *Table 2*). The FLD+ group also had a higher SDS, 2.56 vs. 1.63, *P* < 0.001. SRS tended to be lower and SSS higher in the FLD+ group, although this did not reach statistical significance. Prevalence of ischaemia (SDS ≥ 3) was higher in FLD+ than in FLD− (40.4 vs. 20.4%, *P* < 0.001). Mean SDS was statistically significantly higher in FLD+ patients regardless of their CAD status, *P* < 0.001, i.e. FLD+ patients with previously diagnosed CAD had a higher SDS than FLD− patients with CAD; the same was observed for patients without CAD. Ischaemia% showed similar results, although significance was only reached when comparing the FLD+ and FLD− with CAD (*P* = 0.003) (*Figure 2*).

**Table 2:** Quantitative myocardial perfusion and volume parameters

	Pooled population	FLD−	FLD+	*P*-value (FLD− vs. FLF+, age- and sex-adjusted)
Perfusion scores	(*n* = 742)	(*n* = 606)	(*n* = 136)	
Ischaemia%	3.8 ± 2.5	3.6 ± 2.4	4.5 ± 2.6	**0.001**
SDS	1.8 ± 2.1	1.6 ± 2.00	2.7 ± 2.6	**<0**.**001**
SDS ≥ 3	203 (27.4%)	148 (24.4%)	55 (40.4%)	**<0**.**001**
SRS	2.2 ± 4.2	2.3 ± 4.2	2.00 ± 4.1	0.327
SSS	4.2 ± 5.2	4.0 ± 5.1	4.8 ± 5.3	0.164
Volume parameters at rest	(*n* = 730)	(*n* = 597)	(*n* = 133)	
EDV (mL)	109 ± 55	109 ± 58	109 ± 44	0.260
ESV (mL)	48 ± 48	48 ± 50	48 ± 37	0.396
EF (%)	62 ± 16	62 ± 16	61 ± 15	0.764

Values are mean ± SD or number of cases (%). Statistically significant *P*-values in bold.

In age- and sex-adjusted comparisons between the groups, differences in volume parameters, such as EDV, ESV, and EF, did not exhibit statistical significance. Patients with FLD+ had a 2.10-fold (95% CI 1.42–3.10, *P* < 0.001) greater risk for ischaemia compared with their FLD− counterparts (Model 1). When age, sex, overweight/obesity, hypertension, dyslipidaemia, Type 2 diabetes, and previous diagnosis of CAD were taken into account as potential confounders, FLD was found to be independently associated with myocardial ischaemia (odds ratio 1.70, 95% CI 1.11–2.58, *P* = 0.014; Model 2 i.e. after confounder adjustment using propensity score) (*Table 2*).

## Discussion

The main finding of this study is that clinically significant FLD associated with increased risk for myocardial ischaemia can be detected using the LDCT belonging to the MPI SPECT/CT protocol. FLD is independently associated with myocardial ischaemia. Also, those with previously diagnosed CAD and additional FLD had more severe ischaemia than their non-FLD counterparts. Furthermore, the association was independent of common risk factors for CVD, such as overweight/obesity, Type 2 diabetes, hypertension, dyslipidaemia, age, or sex.

### FLD and CVD risk factors

FLD is strongly associated with metabolic syndrome and its components, such as insulin resistance, obesity, dyslipidaemia, and hypertension.^24^ All these, except dyslipidaemia, displayed statistical significance in our study, with the FLD+ group having a higher prevalence of the comorbidities. This was further validated by the absence of a significant difference in the prevalence of cholesterol medication. Even though FLD and its comorbidities are independently associated with functional and structural changes in the heart, the mechanisms and extent of FLD as a CVD risk factor are unclear.^24^

### FLD and cardiac functional modification

Conflicting evidence has been published on the association between FLD and left ventricular function. Some studies have shown an association between FLD and LVEF,^12–14^ while many others have reported results similar to ours and have not found a significant association between FLD and LVEF.^15–18^

Left ventricle volume parameters did not exhibit any significant differences between the two groups in ECG-gated rest imaging. Similar results have been reported in previous studies, which have failed to observe an effect by FLD on EDV or ESV^25–27^ or on other parameters such as stroke volume or EF.^18,28^ Systolic blood pressure was identical between the two groups, while diastolic blood pressure was significantly higher in the FLD+ group even after adjusting for age and sex. Since previous studies have shown that FLD increases both systolic and diastolic blood pressure,^17^ we believe that the higher prevalence of anti-hypertensive medications in the FLD+ group affected the significance of blood pressure measurements.

### FLD, CAD, and myocardial ischaemia

Our findings indicate that FLD is associated with increased ischaemia, independent of other risk factors. This is in line with the findings of other studies reporting that FLD is associated with causes of myocardial ischaemia, such as increased coronary and carotid atherosclerosis, intima media thickness, and calcification of valves.^9,11,29,30^ Any or all of these could be behind the increase in ischaemia in patients with FLD as seen in our study. The increase in ischaemia is further validated as FLD causes a decrease in myocardial blood flow, during both rest and stress.^31^ Nakamori *et al.*^26^ concluded that FLD was associated with a decrease in myocardial perfusion reserve.

A study by Ren *et al.*^32^ examined the association between FLD and myocardial ischaemia. Similarly to our study, they quantified FLD using an L/S of <1 where, in CT MPI, a global myocardial blood flow of <100 mL/100 mL/min reflected myocardial ischaemia. FLD exhibited a hazard ratio of 2.4 for ischaemia, while in our study, the corresponding risk was 1.78-fold.

Although ischaemia was more prevalent in the FLD+ than in the FLD− group, previously diagnosed CAD was less common in the FLD+ group, though this difference was not statistically significant. About 40% of the FLD+ group had myocardial ischaemia, while just under a quarter (24.4%) of the FLD− group had myocardial ischaemia.^33^ Those with CAD and FLD had statistically significantly higher SDS and ischaemia% than those with CAD but without FLD (*Figure 2*). Even in the absence of previous CAD diagnosis, FLD+ patients were more prone to the occurrence of ischaemia.

### Use of CT in diagnosis of FLD

Our findings for the association of FLD with myocardial ischaemia combined with the findings of previous studies would indicate a need for FLD diagnosis in clinical practice. This is highlighted by the results of Ichikawa *et al.*^34^ indicating that simultaneous evaluation of hepatic steatosis in patients with suspected CAD provides a more accurate predictor of major adverse cardiovascular events and those of Meyersohn *et al.*^35^ showing that hepatic steatosis in patients without CAD is associated with a 1.69-fold increased risk for major adverse cardiovascular endpoints.

Several different methods of diagnosing FLD from CT images with either absolute HU values or HU value ratios have been proposed, with different cut-off points. In this study, the L/S ratio <1.0 was used as a criterion for FLD. When comparing typically used diagnostic doses with those in low-dose scans, Husarik *et al*.^36^ demonstrated liver attenuation to be comparable between the two, even with low-dose scans having significantly higher image noise. While another study did show a significant difference between individual measurements when the dose was reduced, the difference for L/S was negligible even with a substantially reduced dose.^37^

Even though proton density fat fraction in magnetic resonance imaging is claimed to have the best combination of accuracy, precision, and reproducibility to detect hepatic steatosis,^38^ LDCT-based quantification is still a viable option and is much more accessible. Further, CT quantification of hepatic steatosis was shown to correlate linearly with proton density fat fraction.^38^

Our study does have its limitations, for instance individuals with FLD+ are more prone to be overweight and obese, and this increases the likelihood of artefacts in the CT images, which can affect the accuracy of FLD quantification. Another limitation of this article is the lack of coronary calcification data. Only non-gated LDCTs were available to us, and determining the exact calcium score would only be possible from ECG-gated imaging. However, in clinical practice along with fatty liver, another important point to consider in LDCT would be coronary calcification, which can be visually evaluated from non-gated LDCT images.

In conclusion, even though we did not use the most accurate method for diagnosing FLD, we were able to note an association between FLD and decreased myocardial perfusion. This would indicate that, although it might miss some individuals with low-grade steatosis, this fast and accessible method of calculating the L/S ratio from LDCT may be adequate. Furthermore, in cases of doubt, FLD diagnosis could easily be confirmed using ultrasound and blood tests. Since imaging of the cardiovascular system is often performed using CT, we would use the same images to diagnose FLD as a free by-product, as the liver and spleen are often visible on the lowest slices of the images. Assessment of FLD by low-dose CT offers personalized risk assessment to optimize therapy allocation. A co-diagnosis of FLD could be used to highlight the importance of lifestyle modifications, such as diet, weight loss, and abstaining from alcohol consumption.^39^ Guidelines also recommend that pharmacological treatment should focus on cardiovascular risk factors, such as dyslipidaemia.

## Data Availability

Decreased L/S in LDCT as a biomarker of FLD reflects risk for myocardial ischaemia. The data underlying this article cannot be shared publicly due to permission to use this data is restricted by the General Data Protection Regulation (EU) 2016/679, and the Finnish authority (FINDATA) has defined the users and limited operating environment (THL/5431/14.02.00/2020). The outputs of statistical analyses are available by request to the corresponding author.

## Lead author biography

Antti Hokkanen is a final year medical student in University College Cork, Ireland. He is also a doctorate student under the Department of Clinical Physiology and Nuclear Medicine in the University of Eastern Finland, Finland.
